# Visual inspection with acetic acid and colposcopy: screening of cervical cancer in resource-limited healthcare settings

**DOI:** 10.3389/fgwh.2025.1684205

**Published:** 2025-12-12

**Authors:** Hasin Anupama Azhari, Masum Chawdhury, Farzana Islam, Golam Abu Zakaria, Koustuv Dalal, Hasan Mahmud Reza

**Affiliations:** 1 Institute of Natural Sciences, United International University (UIU), Dhaka, Bangladesh; 2 Alo Bhubon Trust (Alo-BT), Dhaka, Bangladesh; 3 Department of Applied Bio Sciences and Process Engineering, Anhalt University of Applied Sciences, Köthen, Germany; 4 Division of Public Health Science, Institute of Health Sciences, Mid Sweden University, Sundsvall, Sweden; 5 Department of Pharmaceutical Sciences, North South University (NSU), Dhaka, Bangladesh

**Keywords:** colposcopy, cervical cancer, VIA, screening, Bangladesh

## Abstract

**Background:**

This study was conducted to assess the effectiveness of visual inspection with acetic acid (VIA) followed by colposcopy for cervical cancer screening. Like many low- and middle-income countries (LMICs), Bangladesh struggles with inadequate cervical cancer screening and diagnostic facilities, as well as a shortage of cytopathologists and histopathologists in remote rural areas. Human papillomavirus (HPV) testing has not yet been implemented effectively in Bangladesh, and cytology (Pap smear) is a costly procedure. The current study performed VIA and colposcopy on apparently healthy adult women, primarily to screen for cervical lesions and, secondarily, to identify associated risk factors.

**Methods:**

This cross-sectional study was conducted in a remote rural health center in Bangladesh using a straightforward and affordable approach: VIA followed by colposcopy. This facility-based, cross-sectional study included 384 married women aged between 18 and 65 years recruited after field-level awareness on cervical cancer prevention.

**Results:**

Out of 384 women tested, 247 (64.3%) were adults, 85 (22.1%) were middle-aged, 33 (8.6%) were older, and only 19 (4.9%) were young adults. The study found that more than one-third of the participants (39.1%) engaged in sexual activities without using condoms. A total of 20 participants tested VIA-positive (5.2%), of whom 60% were confirmed by colposcopy. The chi-squared test identified multiple sexual exposures without condom use as a significant risk factor for cervical cancer. All double-positive cases (*n* = 12) received treatment; 7 (58.3%) underwent thermocoagulation (heat-based ablation), and 5 (41.7%) received a loop electrosurgical excision procedure (LEEP) at referral hospitals.

**Conclusion:**

We propose that, to achieve Sustainable Development Goals 3.7 and 3.8, VIA followed by colposcopy is suitable for screening cervical cancer in rural areas of Bangladesh and other LMICs, where screening techniques such as Pap smear and HPV tests are not yet widely available and accessible.

## 1 Introduction

Cervical cancer is a common but preventable malignancy in women. The increasing incidence of cervical cancer is becoming a significant burden on our population. The primary etiological factor for cervical cancer is the persistent infection of one of the 13 carcinogenic genotypes of human papillomavirus (HPV), namely, genotypes 16, 18, 31, 33, 35, 39, 45, 51, 52, 56, 58, 59, and 68 (1–3). Approximately 70% of cervical cancer cases are associated with infection by HPV genotypes 16 and 18 (4). The nonavalent vaccine is highly promising as it possesses the potential to increase the prevention of cervical cancer by approximately 70% to 90% (5). In Bangladesh, the HPV vaccination campaign that reached 5.6 million adolescent girls (93% of those aged 10–14, single dose), including those from the most marginalized communities, is an essential step in reducing the incidence of cervical cancer in women (6). However, in low- and middle-income countries (LMICs) such as Bangladesh, the bivalent and quadrivalent vaccines are more common, covering HPV types 16 and 18 (7). Moreover, the HPV 16/18 vaccines have prevented the majority of invasive cervical cancer with a preventive rate of 66.2%. Additionally, the nonavalent vaccine offered further prevention ranging from 4.2%–18.3% (8).

Cervical cancer ranks as the fourth most common cancer among women globally, with roughly 660,000 new cases and approximately 350,000 deaths recorded in 2022. LMICs experience the highest incidence and mortality rates of cervical cancer (9, 10). The infection is most common among sexually active young women aged between 18 and 30 years, with a marked decline in prevalence after age 30. Although cervical cancer is more common in women over 35, the infection often begins earlier and can progress slowly to cancer over time (11). Based on the Surveillance, Epidemiology, and End Results (SEER) data from 2010 to 2014, the median age at diagnosis of cervical cancer is 50, with most new cases occurring between ages 35 and 54; however, approximately 20% of women diagnosed are over 65 years old (12). Over the past three decades, the percentage of young women affected by cervical cancer in Bangladesh has significantly increased, with rates ranging from 10% to 40% (13). Cervical cancer is the second most common cancer among women in Bangladesh.

Bangladesh has a dense population of over 165 million, nearly half of whom are female, with most living in rural areas (2022 census) (14). Despite the absence of a comprehensive national cancer registry, data from GOLOBOCAN 2020 reveal that Bangladesh has an age-standardized incidence rate of approximately 10.6 per 100,000 women and a mortality rate of approximately 6.7 per 100,000 women for cervical cancer (10, 15–17). Previous studies have reported that low-income individuals and rural or remote populations with limited access to healthcare are less likely to undergo cervical cancer screening, with fewer than 10% of eligible women in Bangladesh screened by any method (18, 19). According to the International Agency for Research on Cancer (IARC), more than 50 million women in Bangladesh are at risk of cervical cancer, resulting in an estimated 17,686 new cases and 10,362 fatalities each year (20). Similar to other developing countries, the high mortality rate in Bangladesh is due to poor awareness of symptoms, risk factors, screening, and prevention (21). The “National Strategy for Cervical Cancer Prevention and Control,” spanning from 2017 to 2022, was introduced by the Ministry of Health and Family Welfare, Bangladesh, to align the agenda and activities among stakeholders for a concerted effort in combating cervical cancer in the country (22).

Although the immune system naturally clears approximately 90% of HPV infections, persistent infections increase the risk of developing cervical cancer by promoting the formation of precancerous lesions. These lesions, which can progress to cervical cancer within approximately 10 years, are asymptomatic and detectable only through cervical screening. Timely detection can significantly reduce the risk of developing cervical cancer (11).

Screening helps prevent cervical cancer by identifying high-grade lesions [Cervical Intraepithelial Neoplasia (CIN) grades; CIN2+] that can be treated before turning invasive. Various organizations, including the United States Preventive Services Task Force (USPSTF), American Cancer Society (ACS), American College of Obstetricians and Gynecologists (ACOG), National Comprehensive Cancer Network (NCCN), and Centers for Disease Control and Prevention (CDC), recommend regular screening for eligible age groups. Increasingly, countries are transitioning from conventional cytology, or Pap smear tests, which examine cervical cells, to HPV tests, which detect the presence of the virus, due to the latter's higher sensitivity for detecting CIN2+. Although HPV testing is the most effective screening tool for cervical cancer, it is costly. In contrast, more affordable and accessible options such as visual inspection with acetic acid (VIA) and colposcopy could be used as screening tools for cervical cancer, especially beneficial for women in rural areas of Bangladesh and other LMICs (23, 24).

VIA is an inexpensive and straightforward screening method that requires only a speculum, a light source, and 5% acetic acid. Meta-analysis data indicate that it offers moderate performance, with a sensitivity of approximately 72% (95% CI: 64%–79%) and a specificity of approximately 75% (95% CI: 57%–87%) (25). Moreover, it is easy to perform (healthcare providers can be quickly trained), provides immediate results, and allows for prompt follow-up evaluation or treatment, making it highly practical for use in resource-limited settings (26). However, a better outcome is achieved when VIA is combined with colposcopy. In fact, a colposcope with Lugol's iodine allows clinicians to see magnified epithelial and vascular patterns and iodine uptake—the major signs of high-grade lesions.

Considering this, population-based cervical cancer screening was initiated in Bangladesh in 2004 as a pilot study and in 2005 as a national program. The Bangladeshi government has established VIA and colposcopy centers, trained skilled personnel, developed an effective referral system, and implemented a practical monitoring system (24). Different studies show that HPV screening significantly reduces the incidence of advanced cervical cancer and mortality. Methods such as cytology (Pap smear) and HPV testing are effective in identifying high-grade lesions, whereas VIA is less reliable for detecting CIN2+ lesions (26, 27).

However, in a country like Bangladesh, where >80% of people reside in rural areas and face widespread poverty, most of them cannot afford expensive tests such as HPV screening. In addition, in rural areas, there is a low-resource laboratory setup and no trained workers for HPV screening. Therefore, in this study, we aimed to perform VIA and colposcopy on apparently healthy adult women in Boalia Union, Naogaon District of Bangladesh, primarily to screen for cervical lesions and, secondarily, to identify associated risk factors. The study outcomes will also help plan a comprehensive study further. This study assessed the effectiveness of VIA- and colposcopy-based screening for cervical cancer in a resource-limited rural area of Bangladesh, identified associated risk factors, and explored the feasibility of timely intervention.

## 2 Methods

### 2.1 Study design and setting

This facility-based, cross-sectional study was conducted in Boalia Union, Naogaon District of Bangladesh. Naogaon, a district in northern Bangladesh, has 11 smaller administrative regions called upazilas. Naogaon Sadar Upazila is one of these regions and is made up of 12 smaller administrative units called unions, including Boalia. The total population of Boalia Union is 16,393, comprising 8,397 males and 7,996 females. Among the population, 27% were aged between 30 and 49 years. The literacy rate of this population is 51.1% (Community Report, Naogaon).

Screening procedures were conducted at the Rahima Baniz Health Care Centre (RBHC), Boalia Union, Naogaon. Treatment and follow-up were conducted at the Naogaon Sadar Hospital and Rajshahi Medical College Hospital (RMCH), Rajshahi. Examinations were conducted on healthy married women aged between 18 and 65 years who provided written consent. We included females aged 18 years old because in Bangladesh, many have experienced their first coitus before 18 due to child marriage, as evidenced in several published papers (25). The consent form included information about the VIA test, colposcopy (Gynocular, Gynius Plus AB, Sweden, SN: GY221209) examination, and accompanying procedures such as photography, and potential complications that may occur during screening. Eligible participants were non-pregnant, had an intact uterus, and no history of CIN or cancer, and were willing to undergo screening. The study was conducted from 01 June 2022 to 31 May 2023.

The sample size for this study was calculated as follows:

$$n=Z^{2}P\;(1-P)/d^{2}$$

*n*: total number of participants in this study

*Z*: level of confidence desired for the estimate (e.g., 1.96 for a 95% confidence level).

*P*: estimated proportion of the population (here, *P* = 20.6% or 0.2)

1 − *P*: complement of the estimated proportion and represents the proportion of the population that does not possess the characteristic

*d*: desired margin of error(here, *d* = 5%)

The formula essentially states that the required sample size (*n*) is influenced by the desired level of confidence (*Z*), the estimated population proportion (*P*), its complement (1 − *P*), and the desired margin of error (*d*). Based on this calculation, the required number of participants was 375. However, we conducted this study among 384 participants, which is applicable for a large population size.

The ages of the subjects were categorized into four sequential groups: young adults (18–25 years), adults (26–44 years), middle-aged adults (45–59 years), and older-aged adults (60 years and above).

### 2.2 Obtaining a relevant medical history

Prior to the procedure, relevant obstetric and gynecological history, including the number of pregnancies, gynecological diseases, sexual behavior, cervical cancer screening, and psychosocial assessment, was obtained and documented. This information guided the interpretation of VIA and colposcopy findings.

### 2.3 VIA and colposcopy screening procedure

The trained healthcare providers of RBHC went door-to-door to raise awareness about cervical cancer and helped participants in completing survey forms. Interested women were invited to RBHC, where the screening was conducted. Before testing, participants were thoroughly informed about the screening procedure, and written informed consent was obtained. Women were placed on a couch for inspection in a relaxed state after being assured that the procedure would be painless. A warm, sterile vaginal Cusco's speculum was gently inserted to view the cervix, and a cotton swab soaked with 5% acetic acid was gently applied. The senior nurse then examined the vagina for any abnormalities with the naked eye. Women who were VIA-positive underwent an examination by colposcope Lugol's application iodine (VILI) to detect cervical epithelium, highlighting glycogen-rich normal tissue and identifying abnormal vascular patterns associated with dysplastic or precancerous lesions. VIA-positive cases are not always subjected to treatment, as atypical squamous cells of undetermined significance (ASCUS) represent minor cervical cytologic abnormalities that warrant follow-up with repeat testing to assess the risk of underlying precancerous changes (28). Hence, the combined method allowed more accurate diagnosis than VIA alone and enabled remote assessment by gynecologists (29). In addition, this configuration allowed gynecologists to remotely record, store, and transmit photos and videos of the cervix for prompt detection of precancerous and malignant lesions in accordance with the WHO guidelines (16).

### 2.4 Treatment and follow-up

Women with double-positive results were referred to RMCH and Naogaon Sadar Hospital for treatment. In these facilities, the patients underwent thermocoagulation, thermoablation, and loop electrosurgical excision procedure (LEEP) as required. Thermoablation is recommended for women with small, clearly visible lesions that lie entirely on the cervix and when the squamocolumnar junction is completely visible. Conversely, LEEP is recommended when lesions are big, extend into the endocervical canal, or necessitate histological evaluation of removed tissue (30). Patients were monitored for 3–6 months after therapy, and early results show that CINs were successfully eliminated in the majority of cases. Recurrence is being monitored for an extended period of time.

### 2.5 Data collection and statistical analysis

The data collection form had three sections: age, age at marriage, age at first delivery, number of children, occupation, and educational background; multiple sexual partners without condom use and a positive family history of cancer; and VIA and colposcopy test results. Data were analyzed using SPSS 23.

## 3 Results

Table 1 presents the distribution of patients across different age groups and relevant characteristics. The majority of participants were in the adult group (64.3%), with a mean age of 42.7 ± 10.7 years. Nearly 95% of participants got married at a young adult age (18–25) with a mean marital age of 19.9 ± 1.9, and only a small proportion (5.2%) got married at an adult age (26–44). The majority (97.4%) of childbirths occurred in the young adult group. The most common number of children was 2–4 (36.4%). In this study, we found that 14% of women engaged in sexual activities with multiple partners and 39% engaged in sexual activities without condoms.

**Table 1:** Age distribution, number of children, and VIA test results (*n* = 384).

Age (years)	Number of cases (*n*)	Percentage (%)	Age (mean ± SD)
Young adult (18–25)	19	4.9	42.7 ± 10.7
Adult (26–44)	247	64.3	
Middle-aged adult (45–59)	85	22.1	
Older-age adult (60 and above)	33	8.6	
Age at marriage			
Young adult (18–25)	364	94.8	19.9 ± 1.9
Adult (26–44)	20	5.2	
Age at first delivery			
Young adult (18–25)	374	97.4	
Adult (26–44)	5	1.3	
No baby	5	1.3	
Number of children			
1	108	28.1	
2–4	140	36.4	
>4	131	34.1	
None	5	1.3	
VIA test results			
Positive	20	5.2	
Negative	364	94.8	

**Figure 1:**
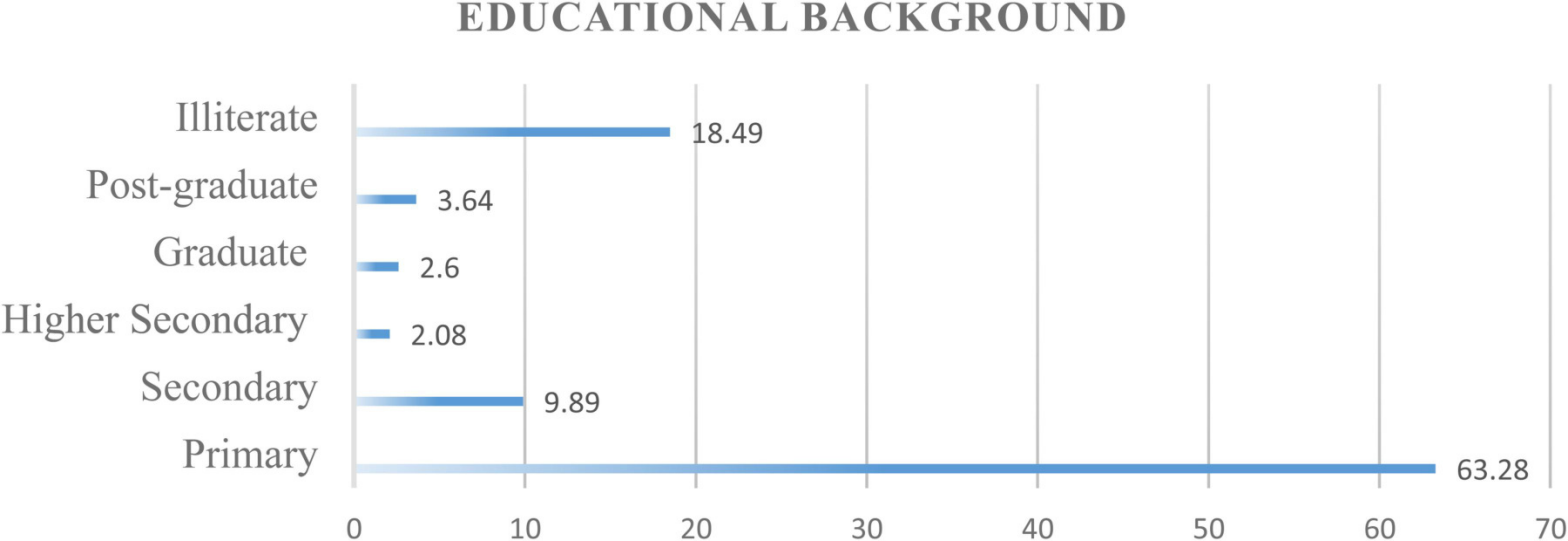
Educational background.

Figure 1 shows a comprehensive overview of educational background and depicts the educational landscape, revealing that 63.3% of women received primary education, 2.1% completed higher secondary, 3.6% completed postgraduate education, and 18.5% remained illiterate.

Table 2 presents the results of the VIA and colposcopy tests, along with the treatment administered to the VIA- and colposcopy-positive patients. Among the 384 participants, 20 were VIA-positive, with the majority (14) in the age group of 26–44 years. Twelve out of the 20 VIA-positive women were further found to be positive by colposcopic examination. All women who were double-positive received either thermocoagulation (seven women) or LEEP (five women).

**Table 2:** Colposcopy results and treatment of selected VIA-positive participants.

Total sample	Age group	VIA-positive (*N* = 384)	Colposcopy confirmed (*n* = 20)	Treatment (*n* = 12)	
				Thermocoagulation (T)	LEEP (L)
384	18–25 years	1	1	1 T	
	26–44 years	14	7	4 T	3 L
	45–59 years	5	4	2 T	2 L
	60+	0	0	–	–
	–	20	12	7	5

## 4 Discussion

This study identified key risk factors and outcomes of cervical cancer screening in a resource-limited setting. VIA-positive cases do not always receive treatment as low-grade lesions such as ASCUS and CIN I may regress spontaneously. These cases are monitored with follow-up, while higher-grade lesions (CIN II+) are referred for treatment (26). Among the 384 women screened, 5.2% were VIA-positive, of whom 60% were confirmed by colposcopy. Notably, 39.1% women reported unprotected sexual activity, which was significantly associated with cervical cancer risk. All confirmed (double-positive) cases received treatment—either thermocoagulation (58.3%) for CIN 2 lesions suitable for outpatient management or LEEP (41.7%) for CIN 3 and carcinoma *in situ*—performed at referral hospitals. These findings underscore the practical value of the VIA test with colposcopy for screening cervical cancer and targeted treatment, as follow-up showed successful clearance of most treated lesions.

The current study underscores the importance of targeted screening and management strategies in mitigating the burden of cervical cancer in Bangladesh using a low-cost screening strategy. The findings of this study shed light on various demographic, behavioral, and clinical factors associated with cervical cancer risk among the studied population in a rural setting. The majority of participants in this study belonged to the adult age group, with a mean age of 42.7 years. A substantial proportion of participants got married at a young adult age (18–25 years), highlighting the early onset of marital relationships within this community. Similarly, the occurrence of childbirths predominantly within the young adult group underscores the importance of reproductive health interventions targeted at younger age cohorts. We also found that the majority of participants only completed primary school education.

This study suggests that there is a strong association between multiple sexual exposures without condom use and VIA positivity. According to Kumar S (33), unprotected sex can lead to cervical cancer by transmitting HPV. In this study, VIA-positive cases were found to be 5.2% (29). Mulmi et al. (29) conducted a study in Nepal that demonstrated a community-level VIA positivity rate of 2.1%, which is slightly lower than the rate found in our study. This may be due to variations in the study population and sites. Their study was a hospital-based cross-sectional descriptive-analytic study involving symptomatic cervical cancer patients, whereas our study was conducted in a remote village involving asymptomatic patients.

Furthermore, the current study identified 20 VIA-positive patients, of whom 12 were both VIA- and colposcopy-positive. These double-positive patients were referred to specialized hospitals for further assessment and treatment. Histological confirmation via biopsy was performed where needed, before definitive treatment. This finding is consistent with a previous study that demonstrated the effectiveness of double tests, such as VIA and colposcopy, in diagnosing cervical lesions (31).

To achieve Sustainable Development Goals 3.7 and 3.8 (sexual and reproductive health and universal health coverage, respectively), using VIA followed by colposcopy to enhance specificity can be used to preliminarily identify cervical cancer cases in rural areas in LMICs where women receive minimal healthcare services for multiple reasons. For detecting CIN2+ lesions, VIA demonstrated a sensitivity of 86.6% and a specificity of 71.6% with corresponding positive and negative predictive values of 30.3% and 97.4%. Notably, specificity improved with increasing age, whereas sensitivity remained relatively unchanged (32). However, this method is highly cost-effective and suitable for implementation in rural Bangladesh and other LMICs, where screening techniques such as Pap smear (cytology) and HPV testing are not yet widely available and accessible.

## 5 Limitations

This study has a few limitations. The sample size is small, as it was challenging to persuade the majority of women to attend screening due to societal norms, superstitions, and regional beliefs despite awareness campaigns. The study was conducted only in a single center (RBHC) because other facilities in Boalia Union do not offer cancer screening services; we believe there are other potential candidates for screening elsewhere. Moreover, participants were placed on a couch during cervical screening with VIA instead of a regular gynecological table with a footrest, which compromised the optimal condition for screening.

## 6 Conclusion

Women in rural Bangladesh are at high risk of cervical cancer. Despite this fact, the majority of women in rural Bangladesh are unaware of cervical cancer. VIA with colposcopy can offer a cost-effective approach for cervical cancer screening in LMICs. In the current study, we were able to help 12 double-positive cases for CIN treatment. For accurate diagnosis of high-grade lesions and cervical cancer, more definitive diagnostic approaches such as HPV testing and histological evaluation are needed. To reduce the financial burden, logistical challenges, and limited access to tertiary hospitals, future research should emphasize community-based education and awareness on cervical cancer and reproductive health, vaccination, and implementation of sustainable screen and treat clinics at the point of care. Integrating these strategies into the national guidelines would help align local practices with WHO recommendations for cost-effective cervical cancer screening and treatment in LMICs

## Data Availability

The raw data supporting the conclusions of this article will be made available by the authors, without undue reservation.
